# Severe Neurocognitive Manifestations in Healthy Young Individuals With COVID-19: A Case Series

**DOI:** 10.1177/11795476251401717

**Published:** 2025-12-17

**Authors:** Akram Farhat, Firas Jaber, Nazih Jaber, Aurélie Fayet

**Affiliations:** 1 Clinical Research Center, Lausanne University Hospital and University of Lausanne, Lausanne, Switzerland; 2 Faculty of Medicine, Beirut Arab University, Beirut, Lebanon; 3 Department of General Medicine, Raee Hospital, Saida, Lebanon

**Keywords:** COVID-19, mental disorders, neurologic manifestations, young adult, case reports

## Abstract

**Background:**

A growing number of patients suffering from neurocognitive symptoms related to COVID-19 were observed as the pandemic progressed. This article addresses severe neurological manifestations in healthy individuals with COVID-19, which were not previously identified in the absence of comorbidities or respiratory symptoms.

**Case presentations:**

The 3 patients, aged between 15 and 27 years old, had no relevant medical history and were not on any chronic treatments. They presented different neurological symptoms (pseudobulbar affect, excessive crying, paracusia, fatigue and sleep problems, anxiety, depression, alexithymia, dementia, aggressive behavior, panic attack, etc) for a period of 2 weeks to 4 months and were treated with different medications. Clomipramine, Zolpidem and Clonazepam were effective in treating these patients.

**Conclusion:**

Even in healthy young individuals with COVID-19, severe neurocognitive manifestations could appear with possible long-term consequences. Further research is needed to expand the knowledge on mechanisms, targeted therapeutic strategies and predictors identifying at-risk groups.

## Background

The coronavirus disease 2019 (COVID-19) pandemic originated in China and spread throughout the world in 2020. The typical clinical symptoms of hospitalized patients were primarily related to the respiratory system. Furthermore, a growing number of patients suffering from neurocognitive deficits related to COVID-19 were observed as the pandemic progressed.

Few studies investigating neurocognitive symptoms in older patients with COVID-19 have been published. The specific related mechanism in these patients remains unclear.^1^ The mechanisms responsible for the neurological manifestations are studied by many researchers.^2^ The neurological complications could be related to systemic effects of the viral infection. The long-term outcomes of COVID-19 remain unknown and cognitive functioning should be taken into consideration for rehabilitation of patients.^3^

In healthy adults with COVID-19, literature shows mild neurological manifestations. In contrast, in comorbid patients with COVID-19, publications addressed moderate to severe neurological symptoms.^1,4,5^ Romagnolo et al identified that severe neurological symptoms in COVID-19 patients were associated with existing comorbidities (arterial hypertension, renal failure and neoplastic diseases), older age and male gender.^6^

To the best of our knowledge, severe neurocognitive manifestations in healthy young individuals with COVID-19 were not previously identified. This article addresses neurological manifestations by presenting different severe symptoms in healthy young individuals with COVID-19.

The novelty of this report lies in the presentation of “*previously healthy individuals*” who developed severe neurocognitive manifestations following COVID-19 infection—symptoms that have not been previously reported in this population. In contrast, existing literature has primarily documented such manifestations only in patients with pre-existing comorbidities, significant respiratory symptoms or advanced age.

## Case Presentations

Three cases are presented in Table 1. These cases were followed up by the same treating physician (general practitioner) who collected the data.

**Table 1.** Characteristics of the Patients.

Characteristics	Case 1	Case 2	Case 3
Age (years)	22	27	15
Sex	Female	Male	Female
Relevant medical history	None	None	None
COVID-19 vaccination	Not vaccinated	Vaccinated (2 doses)	Not vaccinated
Respiratory symptoms	Mild cough	Absent	Absent
Other symptoms	- Alexithymia - Pseudobulbar affect (PBA) - Dementia with severe disorientation and instability that affects the ability to perform everyday tasks - Aggressive behavior with the intention of inflicting damage or other self-harm - Anxiety - Fatigue and sleep problems	- Anxiety and panic attack - Hypochondria - Depression	- Loneliness and autophobia - Paracusia - Anxiety - Acute stress disorder - Social anxiety disorder - Excessive crying
Symptoms onset after infection (days)	10	14	7
Admission	Emergency	Hospitalized	Hospitalized
Improvement time (days)	14	90	120

The MoCA (Montreal Cognitive Assessment) tool was used for cognitive assessment, and the HADS Scale (Hospital Anxiety and Depression) was used for anxiety and depression assessment.

### Case 1

A 22-year-old female tested positive for COVID-19 in January 2023 (mild COVID-19 symptoms). The patient was healthy. She had no relevant medical history (including pertinent psycho-social history, family medical history, identified genetic disorders and relevant past interventions) and was not on any chronic treatments. The patient presented the following symptoms (in the absence of respiratory manifestations), 10 days after infection, for which she was admitted to the emergency unit without being hospitalized:

- Alexithymia (inability to describe emotions)
- Pseudobulbar affect PBA (sudden uncontrollable and inappropriate laughing and crying)
- Dementia with severe disorientation and instability that affects the ability to perform everyday tasks
- Aggressive behavior with the intention of inflicting damage or other self-harm
- Anxiety
- Fatigue and sleep problems

At the emergency unit, an MRI and a CT scan of the brain were performed, and the results did not show any abnormalities, according to the report of the physician. The patient also presented mild cough and fever without additional respiratory symptoms or other complications. She was treated with an antipyretic drug (paracetamol).

The symptoms persisted for 2 weeks before improvement was observed. This case was followed up by the physician for 2 months after clinical improvement, during which no symptoms were reported.

### Case 2

A 27-year-old male tested positive for COVID-19 in January 2023 (mild COVID-19 symptoms). The patient was healthy and had received 2 doses of the COVID-19 vaccine. He had no relevant medical history (including pertinent psycho-social history, family medical history, known genetic disorders or relevant previous interventions) and was not on any chronic treatments. The patient presented the following symptoms (in the absence of respiratory manifestations), 2 weeks after infection, for which he was hospitalized:

- Anxiety and panic attack
- Hypochondria (illness anxiety disorder or health anxiety)
- Depression

The patient was treated with different medications (Fluoxetine 20 mg, Escitalopram 10 mg, Olanzapine) between January 2023 and March 2023, without any improvement. Subsequently, the patient was treated with clomipramine 25 mg which was effective. The patient exhibited good adherence and favorable treatment tolerability with no significant adverse events (patient self-report and clinical assessment). The improvement was observed in April 2023. Clomipramine 25 mg was prescribed for 6 months. This case was regularly followed up by the physician for 2 months post-treatment, during which no symptoms were reported.

### Case 3

A 15-year-old female tested positive for COVID-19 in December 2022 (mild COVID-19 symptoms). The patient was healthy. She had no relevant medical history (including pertinent psycho-social history, family medical history, known genetic disorders or relevant previous interventions) and was not on any chronic treatments. The patient presented the following symptoms (in the absence of respiratory manifestations), 1 week after infection, for which she was hospitalized:

- Loneliness and autophobia
- Paracusia (auditory hallucination)
- Anxiety
- Acute stress disorder
- Social anxiety disorder (social phobia)
- Excessive crying

At hospital, an MRI of the brain was performed along with different other tests (among which CBC, hepatic enzyme and creatinine), and the results did not show any abnormalities, according to the report of the physician. Schizophrenia was suspected.

The patient was treated with Risperidone for 2 months, without improvement. Subsequently, the patient was prescribed Zolpidem and Clonazepam, which were effective. The patient exhibited good adherence and favorable treatment tolerability with no significant adverse events (patient self-report and clinical assessment). The improvement was observed in April 2023. This case was followed up by the physician for 2 months post-treatment, during which no symptoms were reported.

## Discussion

This article reported severe neurocognitive manifestations in healthy young individuals with COVID-19 (alexithymia, pseudobulbar affect, dementia, aggressive behavior, fatigue and sleep problems, anxiety and panic attack, hypochondria, depression, loneliness and autophobia, paracusia, acute stress disorder, social anxiety disorder, excessive crying), notably in the absence of respiratory symptoms or pre-existing comorbidities. The 3 reported cases, with no relevant medical history and no chronic medication use, suffered from severe neurological symptoms related to the viral infection, for a period of 2 weeks to 4 months.

The incidence of neurocognitive manifestations among COVID-19 patients could be related to many factors. Evidence suggests that patients with severe COVID-19 might have a hypercytokinaemia, a cytokine storm syndrome, observed in some infections, which causes severe inflammation and other reactions.^2,7^ A strong immune response to COVID-19 leading to a chronic inflammation could contribute to long COVID-19 symptoms that persist several weeks after infection.^8^ Other biological, genetic, psychological and environmental factors could be related to severe manifestations. A *Letter to the Editor* mentioned that vitamin D might prevent neurocognitive complications in COVID-19 patients by reducing the risk of cytokine storm.^9^ The specific related mechanism remains unclear.^1^

Contrary to our cases of “severe” neurological manifestations in “healthy young individuals”, publications addressed either mild symptoms in COVID-19 patients, or moderate to severe symptoms in elderly patients with multimorbidities. Solomon et al reported mild neuropathological findings in COVID-19 older patients (median age of 62 years), with different comorbidities (diabetes mellitus, hypertension, cardiovascular disease, hyperlipidemia) presenting confusional state, headache, decreased taste and myalgia.^4^ Whiteside et al reported moderate to severe manifestations in 3 COVID-19 older patients (aged between 62 and 75 years old) with different comorbidities (diabetes, kidney diseases, hypertension, hyperlipidemia) presenting neurological manifestations among which cognitive impairment, anxiety, depressive symptoms, delirium, less structured memory tasks and emotional distress.^1^ Other studies found a significant relationship between cognitive function and gastrointestinal symptoms;^10^ however, such complaints were absent in our 3 patients. Moreover, a meta-analysis concluded that mild cognitive deficits were associated with COVID-19 infection but failed to observe clinically meaningful cognitive impairments in people with COVID-19 without severe comorbidities.^11^

This is the first article presenting severe neurocognitive manifestations in previously healthy young individuals with COVID-19. However, this report has some limitations that should be considered. First, there was no long-term follow-up. Additionally, our sample size limits the ability to further explore potential associations with cognitive functioning.

## Conclusion

Severe neurocognitive manifestations in COVID-19 patients could appear even in healthy young individuals not suffering from respiratory symptoms, pre-existing comorbidities or advanced age. The related health concern remains the unpredictability of the severe symptoms in individuals who were not previously considered at risk. Further research is needed to expand the knowledge on mechanisms, targeted therapeutic strategies^12 -18^ and predictors identifying at-risk groups.
